# Exploration of Therapeutic Strategies of Herbal Prescriptions for Carbuncle Treatment to Suggest Modern Approaches to Inflammatory Bowel Disease: Cluster and Network Analyses of the Book «*Liu Juan Zi Gui Yi Fang*»

**DOI:** 10.3390/healthcare12151499

**Published:** 2024-07-28

**Authors:** Dasol Park, Heonyoung Jeong, Jungtae Leem

**Affiliations:** 1 Department of Diagnostics, College of Korean Medicine, Wonkwang University, 460, Iksan-daero, Sin-dong, Iksan 54538, Jeollabuk-do, Republic of Korea; mare0927@wku.ac.kr; 2 Department of Medical Classics, College of Korean Medicine, Wonkwang University, 460, Iksan-daero, Sin-dong, Iksan 54538, Jeollabuk-do, Republic of Korea; jikjae@wonkwang.ac.kr; 3 Research Center of Traditional Korean Medicine, College of Korean Medicine, Wonkwang University, 460, Iksan-daero, Sin-dong, Iksan 54538, Jeollabuk-do, Republic of Korea

**Keywords:** abscess and carbuncle, cluster analysis, data mining, inflammatory bowel disease, *Liu Juan Zi Gui Yi Fang*, network analysis, prescription rule, traditional herbal medicine

## Abstract

Inflammatory bowel disease (IBD) treatments in East Asian traditional medicine (EATM) originate from principles for treating abscesses and carbuncles. Understanding the therapeutic principles of *Liu Juan Zi Gui Yi Fang* (*GYF*) is essential for optimizing EATM treatment strategies for IBD, but quantitative analysis is lacking. This study aims to extract quantitative information on therapeutic strategies from *GYF* and present the EATM conceptual framework for IBD treatment. Oral prescriptions for carbuncles were selected, and their constituent herbs and indications were standardized and tokenized for analysis. An EATM expert group classified prescriptions based on the similarity of herbs and indications. Hierarchical and k-means cluster analyses were performed based on herb similarity. The herb–indication (H-I) network for all prescriptions was constructed. Additionally, H-I subnetworks based on the expert group’s classifications and the k-means clustering results were constructed and compared to identify treatment goals and the herbs used for each goal. The results showed that the treatment focused on abscess status, wound healing, and patient’s recovery capacity, with ‘fever’ and ‘deficiency’ as the main indications addressed by tonifying and anti-inflammatory herbs. The therapeutic principles identified in this study can serve as a foundation for developing future herbal intervention units. Further preclinical and clinical research is needed to validate these findings.

## 1. Introduction

Inflammatory bowel disease (IBD), which includes Crohn’s disease and ulcerative colitis, represents chronic and relapsing inflammatory conditions of the gastrointestinal tract [1]. The proper management of IBD is crucial, as it can lead to irreversible complications such as intestinal strictures, fistulae, and colorectal carcinoma [2]. Despite the reduction in bowel resection surgeries and mortality rates with conventional medications, intolerance or the loss of response to medications, increased susceptibility to infections from long-term immunosuppressive therapy, high medical costs, and high relapse rates still significantly impact the quality of life of many IBD patients [3].

In East Asian traditional medicine (EATM), herbal medicines have been used clinically for IBD treatment, and evidence has been accumulated through clinical and experimental studies on the efficacy and safety of various herbal formulations [4,5]. Herbal medicines, with their multi-component, multi-pathway, and multi-target approach, offer a comprehensive treatment strategy for complex diseases like IBD and may provide novel therapeutic options [6]. However, due to the complexity of herbal medicines and the lack of appropriate methodologies to address this complexity, it remains challenging to identify the most effective formulations or components, despite extensive clinical use and experimental studies [7]. Overcoming this challenge requires a thorough understanding of the theories behind herbal medicine treatment, but their abstract concepts and terminology pose a challenge to effective scientific communication [8].

This study aims to quantitatively extract information on currently applied clinical treatment strategies for IBD from the classical literature *Liu Juan Zi Gui Yi Fang* (*GYF*) and present a conceptual framework for EATM approaches to IBD. In EATM, the disease category of abscesses and carbuncles encompasses not only cutaneous conditions but also acute and chronic inflammatory and infectious diseases of internal organs such as the gastrointestinal tract [9]. *GYF*, the earliest extant literature specializing in surgery, compiled in the late 5th century, is a seminal text on the treatment of abscesses and carbuncles [10]. The clinical approach of EATM to IBD has its roots in the treatment principles and prescriptions for abscesses and carbuncles, and formulations based on *GYF* are still used clinically in modern times [11,12]. However, research that quantitatively analyzes the specific therapeutic rules embedded in *GYF*, such as which herb combinations are applied for particular indications, is lacking. Quantitatively analyzing the prescriptions in *GYF* and their indications can provide an intuitive understanding of current EATM treatment strategies for IBD, support clinical decision making, and aid in hypothesis generation and core herbal formulation selection for future research.

This study employed quantitative data mining methodologies, such as cluster analysis and network analysis, in combination with the knowledge of EATM experts to maximize the usefulness of the extracted information.

## 2. Material and Methods

### 2.1. Selection of Prescriptions for Treating Carbuncles

The data source for this study was the 2014 edition of *GYF* published by Beijing Science Technology Publishing House [13]. To distinguish between prescriptions with the same name but different compositions, we assigned a unique ID number to each prescription based on their order in the book. We then selected oral prescriptions with carbuncle and related symptoms recorded in their therapeutic indications for the final analysis. Prescriptions that could be administered both orally and externally were also included. For the selected prescriptions, we extracted data on their compositions and indications. Dosage information was excluded from the analysis due to the lack of standardized dosage units in the formulations. To facilitate analysis and visualization, each prescription was assigned a unique identifier (ID).

### 2.2. Data Extraction, Standardization, and Tokenization

We standardized the herb names according to the Korea Institute of Oriental Medicine website and the 2015 edition of the Chinese Pharmacopoeia [14]. For the indications of each prescription, three EATM experts (DP, HJ, JL) with more than 15 years of clinical and research experience reached a consensus on dividing and standardizing the semantic units, which were then tokenized. To facilitate analysis and visualization, we assigned unique IDs to each herb and indication. We then converted the information of each prescription into a vector of herbs, using 1 if a specific herb was present and 0 if it was absent. The same method was applied to indications, transforming the information of each prescription into a vector of indications. We used Microsoft Excel (v.16.76, Microsoft Corporation, Redmond, WA, USA) to create these prescription–herb and prescription–indication datasets.

### 2.3. Prescription Clustering Based on EATM Expert Knowledge

Three EATM experts (DP, HJ, JL) clustered the prescriptions into groups by considering the similarity of constituent herbs and indications between each prescription. Each prescription was allowed to be classified into more than one group. When the experts determined that useful prescription rule information could be derived, each prescription group was further divided into subgroups based on the similarity of herbs and indications. For convenience, an artificial group ID was assigned to each prescription group.

### 2.4. Hierarchical and Non-Hierarchical Cluster Analysis

Hierarchical and non-hierarchical cluster analyses were performed based on the similarity of constituent herbs between prescriptions. All analyses and visualizations were performed using RStudio (v.2023.06.0, Posit team. RStudio: Integrated Development Environment for R. Posit Software, PBC, Boston, MA, USA).

#### 2.4.1. Jaccard Index Calculation as a Similarity Measure

To perform the cluster analyses, we calculated the Jaccard coefficient as a similarity index for the constituent herbs between prescriptions [15]. The Jaccard distance served as an indicator of how different each prescription was from others. The Jaccard coefficient and Jaccard distance were calculated using the following formulas:

$$\mathrm{Jaccard}\;\mathrm{coefficient}:\;J\left(X,Y\right)=\frac{\left|X\cap Y\right|}{\left|X\cup Y\right|}=\frac{\left|X\cap Y\right|}{\left|X\right|+\left|Y\right|-|X\cap Y|}$$

$$\mathrm{Jaccard}\;\mathrm{distance}:\;d_{jaccard}\left(X,Y\right)=1-J(X,Y)$$

*X* and *Y* represent sets that express the presence or absence of constituent herbs in two different arbitrary prescriptions. $J\left(X,Y\right)$ denotes the Jaccard coefficient between two distinct prescriptions, calculated as the ratio of herbs commonly included in all herbs used in both prescriptions. The Jaccard coefficient indicates the degree of similarity, with values closer to 1 representing higher similarity and values closer to 0 representing lower similarity. A Jaccard distance value closer to 1 signifies that two prescriptions have lower similarity and are more distantly related in the analysis, while a value closer to 0 indicates higher similarity and closer proximity between the prescriptions.

#### 2.4.2. Hierarchical Cluster Analysis

The hierarchical cluster analysis was performed using the ‘stats package’. The results of the hierarchical cluster analysis were visualized as a dendrogram using the ‘dendextend package’, which placed similar prescriptions on closer branches [16].

#### 2.4.3. k-Means Cluster Analysis

k-Means cluster analysis was performed to partition the prescriptions into separate groups [17]. The results of the k-means algorithm can be sensitive to the number of clusters, which must be specified before the analysis, and the initial positions of each cluster’s centroid.

To assess the impact of these factors on the results, we conducted additional sensitivity analyses by varying the number of clusters and initial centroid positions. The position of the initial centroids was controlled using random seeds. We calculated the silhouette index and misclassification percentage to compare the validity of the k-means cluster analyses. The silhouette index is a measure of how well an object fits into its assigned cluster compared to other clusters, with values ranging from −1 to 1, where a higher value indicates a better match to the assigned cluster and a lower value suggests a poor match or that the object is better suited to another cluster [18]. Misclassification was defined as the case where two prescriptions with a Jaccard coefficient of zero were classified into the same cluster. The cluster analysis was conducted using the ‘cluster package’, and the results were visualized using the multidimensional scale function [19].

### 2.5. Construction and Analysis of Heterogeneous Information Network of Herb–Indication (H-I) Relationship

We integrated two datasets of the prescription–herb and prescription–indication relations to generate the herb–indication (H-I) network. In the network visualization, herbs were depicted as circular nodes, whereas indications were square nodes. When a co-appearance between an indication and an herb was identified, they were connected with an edge. The size of each node was set to be proportionate to the frequency of their appearance in the entire prescriptions. The thickness of each edge was set to be proportional to the frequency of H-I co-appearance. The proximity between nodes was adjusted based on the frequency of co-appearance, with frequently co-appeared nodes being nearer to each other.

Furthermore, we generated a subnetwork of H-I relations for each prescription group, which was classified by the EATM expert group and cluster analysis, respectively, and analyzed the major treatment goals and herbs used. The results of the hierarchical cluster analysis were not utilized as they did not create completely separated clusters. The ‘ggplot2 package (v.3.4.4)’ was used for network visualization [20].

## 3. Results

All prescriptions included in *GYF* are presented in Table S1. We selected 43 oral herbal prescriptions for treating carbuncles. Table S2 shows the ID, name, constituent herbs, and original text of therapeutic indications for each selected prescription. The extracted, standardized, and tokenized herbs and indications along with their assigned unique IDs are presented in Tables S3 and S4.

### 3.1. Prescription Clustering by EATM Expert Group

The clustering results of prescriptions by the EATM expert group are presented in Tables S5–S8. The arbitrary names assigned to each prescription group based on common herbs and indications are as follows: ‘Diarrhea treatment prescriptions (E1)’, ‘Abscess treatment prescriptions (E2)’, ‘Da huang-including prescriptions (E3)’, and ‘Tonifying prescriptions (E4)’. E4 was further divided into four subgroups: ‘Huang qi tang fang group (E4-1)’, ‘Sheng di huang tang fang group (E4-2)’, ‘Zhu ye tang fang group (E4-3)’, and ‘Unclassified group (E4-4)’. Dan zhu ye tang fang (P-33) and Huang qi tang fang (P-37) were classified into both E3 and E4 because they contain Da huang (H15) and tonifying herbs. Although Xin yi tang fang (P-54), Zeng sun san fang (P-77), and Qu mai san fang (P-80) include tonifying herbs, the experts did not reach a consensus on their subgroup classification, so they were categorized as ‘Unclassified’.

### 3.2. Results of Hierarchical Cluster Analysis

**Figure 1:**
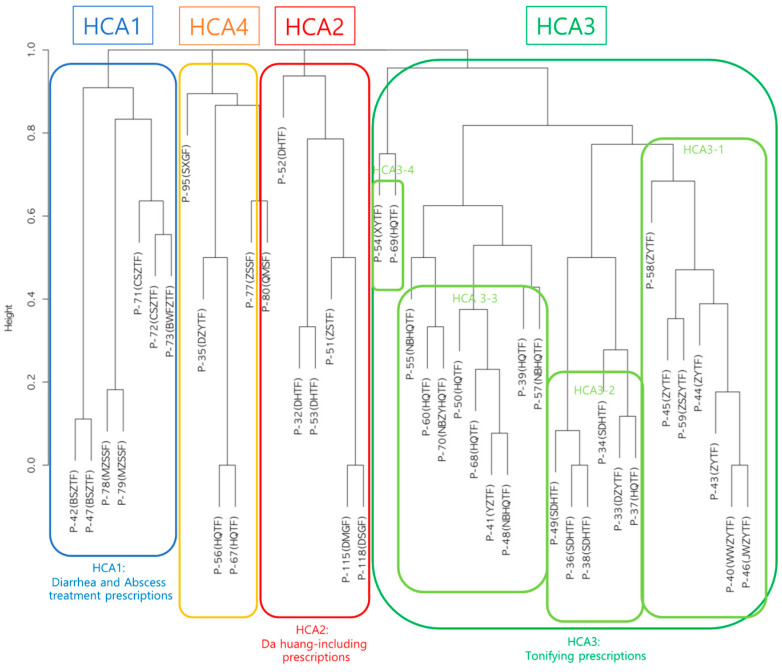
Dendrogram from hierarchical cluster analysis of carbuncle treatment prescriptions based on the similarity of constituent herbs between prescriptions. The specific prescription names and their constituent herbs are listed in Table S2. HCA1, Diarrhea and abscess treatment prescriptions; HCA2, Da huang-including prescriptions; HCA3, Tonifying prescriptions; HCA3-1, Zhu ye tang fang group; HCA3-2, Sheng di huang tang fang group; HCA3-3, Huang qi tang fang group; HCA3-4, Remaining group; HCA4, Remaining prescriptions.

Figure 1 presents the dendrogram visualizing the results of the hierarchical cluster analysis. The dendrogram revealed four main prescription groups, which were assigned the following arbitrary names for convenience: ‘Diarrhea and Abscess treatment prescriptions (HCA1)’, ‘Da huang-including prescriptions (HCA2)’, ‘Tonifying prescriptions (HCA3)’, and ‘Remaining prescriptions (HCA4)’. The HCA3 prescription group was further divided into four subgroups: ‘Zhu ye tang fang group (HCA3-1)’, ‘Sheng di huang tang fang group (HCA3-2)’, ‘Huang qi tang fang group (HCA3-3)’, and ‘Remaining group (HCA3-4)’. The hierarchical cluster analysis results were highly similar to those classified by the EATM expert group. However, ‘Diarrhea treatment prescriptions (E1)’ and ‘Abscess treatment prescriptions (E2)’, which were classified as separate prescription groups by the expert group, were combined into a single prescription group (HCA1) in the hierarchical cluster analysis. Additionally, P-33 and P-37, which contain Da huang, were classified into HCA3, reflecting the high proportion of tonifying herbs in their composition.

### 3.3. Prescription Group Partitioning by k-Means Cluster Analysis

**Figure 2:**
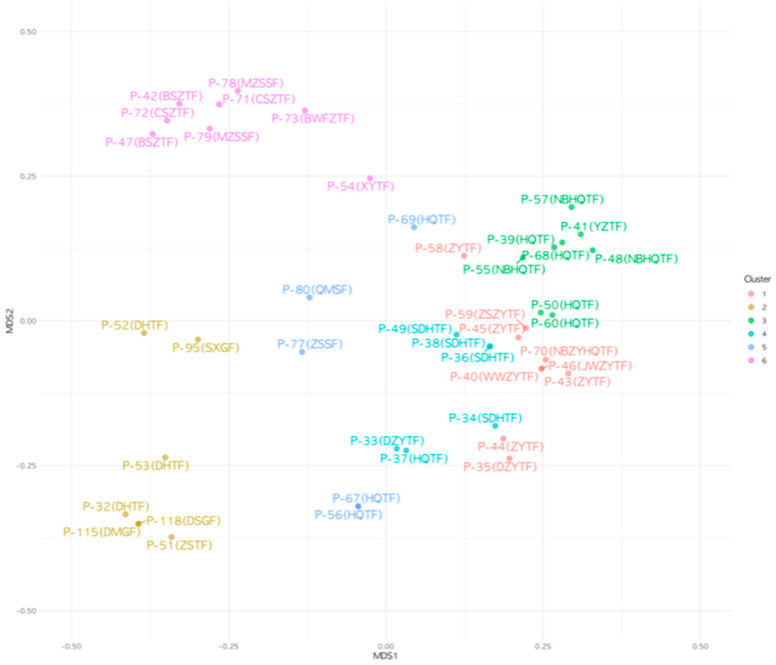
Six clusters of carbuncle treatment prescriptions from k-means cluster analysis with random seed of 12345. The specific prescription names and their constituent herbs are provided in Table S2.

We performed a k-means cluster analysis to generate six clusters. The number of clusters was determined by considering the EATM expert group’s classification and the results of the hierarchical cluster analysis. Figure 2 presents the visualized results of the k-means cluster analysis. With a few exceptions, the k-means cluster analysis results were similar to those of the EATM expert group’s classification and the hierarchical cluster analysis. For convenience, each cluster was named as follows: ‘Diarrhea and Abscess treatment prescriptions (K1)’, ‘Da huang-including prescriptions (K2)’, ‘Huang qi tang fang group (K3)’, ‘Shen di huang tang fang group (K4)’, ‘Zhu ye tang fang group (K5)’, and ‘Others (K6)’.

### 3.4. Sensitivity Analysis of the Impact of the Pre-Specified Number of Clusters on Prescription Clustering by k-Means Cluster Analysis

Table S9 presents the results of prescription clustering by k-means cluster analysis when the number of clusters varied from three to eight, with the initial centroid positions fixed using the same random seed. P-35, P-40, P-43, P-44, P-45, P-46, P-58, P-59, P-58, P-59, and P-70 were consistently classified into the same cluster, which corresponded to the ‘Zhu ye tang fang group (E4-3)’ classified by the EATM expert group. P-41, P-48, P-50, P-55, P-57, P-60, and P-68 were also consistently classified into the same cluster, corresponding to the ‘Huang qi tang fang group (E4-1)’. P-71, P-72, P-73, P-78, and P-79 were also consistently classified into the same cluster, which included prescriptions from ‘Diarrhea treatment prescriptions (E-1)’ and ‘Abscess treatment prescriptions (E-2)’. The consistent classification of these prescriptions into the same clusters reflects their significant distinction from other clusters due to the high similarity of their constituent herbs. The silhouette indices ranged from 0.11 to 0.24, with the lowest values observed when the number of clusters was 3 or 4 and the highest value when it was 7. The misclassification rate was the lowest at 0.22% when the number of clusters was 6. It was 9.52% with 5 clusters, 14.62% with 4 and 3 clusters, and 1.55% with 7 and 8 clusters.

### 3.5. Sensitivity Analysis of the Impact of Initial Centroid Position Changes on Prescription Clustering by k-Means Cluster Analysis

Table S10 presents the results of k-means cluster analysis performed with five different initial centroid positions while keeping the number of clusters fixed at six. In four out of the five cases, prescriptions with high similarity in constituent herbs tended to be consistently classified into the same cluster. However, one case showed a significantly different clustering pattern compared to the others. When the random seed was set to 123, Bai shi zhi tang fang (P-47), which belongs to ‘Diarrhea treatment prescriptions (E1)’, was included in the same cluster as ‘Da huang-including prescriptions (E3)’, resulting in prescription pairs with no common herbs being classified into the same cluster. The silhouette indices ranged from 0.14 to 0.22, with the lowest value observed when the random seed was 123, and the highest value of 0.22 observed for random seeds 12345, 148, and 371. The misclassification rate of the clustering with random seed 123 was relatively high at 9.52%.

### 3.6. Constructing the Herb–Indication Relationship Network for Carbuncle Treatment Prescriptions

**Figure 3:**
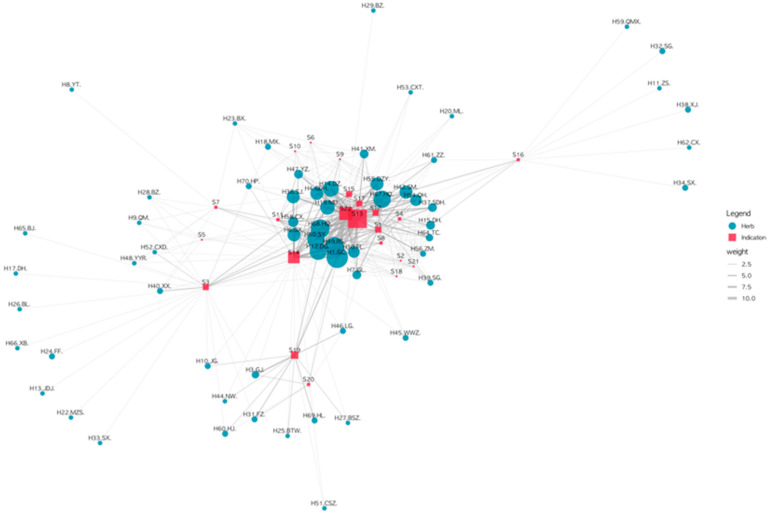
Herb–indication (H-I) network of all carbuncle treatment prescriptions. S1, Thirst; S2, Sign of lumped stools; S3, Abscess; S4, Difficulty in defecation; S5, Pain; S6, Fullness; S7, Abscess unruptured; S8, Vexation/Oppression; S9, Anorexia; S10, Qi reflux; S11, Weak or faint breathing; S12, Difficulty in urination; S13, Fever; S14, Abscess ruptured; S15, Swelling; S16, The early phase of the disease; S17, After laxation; S18, Vomit; S19, Diarrhea; S20, Coldness; S21, Aversion to cold with fever; S22, Deficiency.

The H-I relationship network generated by connecting the datasets of prescriptions and their constituent herbs and indications is presented in Figure 3. In the H-I network, the most frequently observed indication was ‘Fever (S13)’, followed by ‘Deficiency (S22)’. S13 and S22 were highly connected to tonifying herbs such as Huang qi (H68), Ren shen (H49), Gan cao (H1), and Dang gui (H12) on one side and to anti-inflammatory herbs such as Dan zhu ye (H55), Huang qin (H67) and Sheng ma (H42) on the other. The most frequently used herbs in the entire network of carbuncle treatment prescriptions were Gan cao (H1), Ren shen (H49), Huang qi (H68), Dang gui (H12), Shao yao (H50), Gan di huang (H4), Mai dong (H19), Da zao (H14), and Huang qin (H67).

### 3.7. Cluster-Specific H-I Subnetworks

We generated H-I subnetworks for each cluster using the EATM expert group’s classification results and the k-means cluster analysis results. We paired clusters with similar structures and presented them in Tables S11–S16. We utilized six clusters from the EATM expert group’s classification, excluding the ‘Unclassified group (E4-4)’. For the k-means cluster analysis results, we used the outcome that generated six clusters with the lowest misclassification rate in the sensitivity analysis, using the random seed 12345. The three researchers reached a consensus and provided an arbitrary name for convenience for each compared pair using the frequently observed indications, as follows:

‘Diarrhea-Coldness-Abscess-Abscess ruptured’, ‘The early phase of the disease-Difficulty in defecation-Difficulty in urination-Fever’, ‘Abscess ruptured-Deficiency-Fever’, ‘Fever-Deficiency-Thirst’, ‘After laxation-Difficulty in urination-Fever’, and ‘Abscess unruptured-Abscess-Abscess ruptured’. Table 1 summarizes the common herbs and indications for each comparison group.

## 4. Discussion

### 4.1. Summary of Findings

*GYF* is an essential piece of literature that contains foundational clinical information on the principles of carbuncle treatment, which are clinically applied in the treatment of IBD. Considering that the treatment principles and prescriptions of *GYF* are still utilized in modern EATM clinical practice and achieve consistent clinical outcomes, deriving an objective and quantified theoretical framework from *GYF* would serve as a vital resource for designing scientific studies to validate the efficacy of EATM in IBD treatment [11,12].

In general, the carbuncle treatment in the prescriptions recorded in *GYF* is based on the disease phase, with particular emphasis on the status of the abscess, the degree of wound healing, and the patient’s recovery capacity. Other factors that should be considered to determine the prescription include the state of the stool and urine, the presence of fever, the presence of dehydration, and the function of the digestive system. *GYF* predominantly presents ‘fever’ and ‘deficiency’ as the main therapeutic indications for carbuncles. To address these indications, tonifying herbs such as Ren shen (ginseng) and Huang qi (astragalus) are primarily used, while other herbs that can resolve inflammation are combined as adjuvants.

### 4.2. Modern Applications of Herbal Prescriptions for IBD Management in Clinical Practice and Suggestions for Further Research

The cluster and network analyses revealed that the carbuncle treatment prescriptions recorded in *GYF* mainly targeted the status of the abscess, the degree of wound healing, and the patient’s recovery capacity. Microscopic findings in patients with symptomatic and asymptomatic IBD showed endoscopic activity, which was comprehensively described to be associated with prognosis [21]. In this regard, our study results suggest the possibility of applying herbal prescriptions used for IBD treatment in modern EATM clinical practice by subdividing them based on the histopathological findings of the villi and crypts of the intestine and utilizing them for clinical and preclinical efficacy evaluations. In particular, recognizing the patient’s healing capacity along with the frequent utilization of tonifying herbs in carbuncle treatment is one of the key ideas in EATM compared to current pharmacological immunosuppressive strategies for IBD. A previous study has reported the antiulcerogenic activity of Li-Zhong decoction, which is composed of Ren shen, Bai zhi, Gan jiang, and Gan cao, on duodenal ulcers induced by indomethacin in rats [22]. In the study, Li-Zhong decoction markedly reduced pathological events including duodenal hemorrhagic necrosis, inflammatory infiltration, villus destruction, and crypt abscess. Moreover, another study has reported that Huang-Qi-Jian-Zhong-Tang, which is composed of Gui zhi, Huang qi, Gan cao, Shao yao, Sheng jiang, Da zao, and Yi tang, normalized the tissue architecture by increasing villus height and crypt depth in indomethacin-induced duodenal ulcers in rats [23]. However, further research is needed to examine whether the use of such tonifying herbs significantly influences histological healing with short-term and long-term clinical effects, and the results from our quantitative analyses may serve as a foundation for developing herbal units of intervention in future research.

Our study also confirmed that *GYF* emphasizes the importance of considering stool condition and digestive function when determining carbuncle treatment prescriptions. Given that intestinal microbiome dysbiosis significantly influences IBD pathophysiology through its association with intestinal mucosal barrier injury or recovery, the treatment strategies in *GYF* may exert therapeutic effects by influencing the immune system through microbial regulation. Dietary interventions, such as gluten-free diets, have been shown to alleviate intestinal mucosal damage through immunological responses induced by their effects on the gut microbiome [24]. Various studies have demonstrated that herbal medicines can affect IBD through immune modulation via the gut microbiome, either through diverse bioactive compounds or in a probiotic-like manner similar to dietary interventions. For example, berberine found in *Coptis chinensis* have been shown to encourage probiotics development, while *Astragali Radix* has been found to increase beneficial gut bacteria through the glucose degradation process in vitro [25]. Recently, H.L. Mok et al. demonstrated that modified Zhenwu decoction, containing *Codonopsis pilosula*, *Atractylodes macrocephala* Koidz., *Glycyrrhiza uralensis*, *Coptis chinensis*, and *Cornus officinalis*, inhibited proinflammatory macrophage infiltration in the intestinal mucosa of a mouse model of ulcerative colitis by suppressing p38 MAPK activation [26]. These findings suggest that herbal medicines can affect the immune system through microbial regulation, aligning with the treatment strategies in *GYF*. The knowledge extracted from *GYF* relates to key IBD pathophysiological mechanisms, such as intestinal microbial dysregulation, and shows potential for use as conceptual units in developing hypotheses for IBD intervention studies.

### 4.3. Importance of Integrating Expert Insights with Data Mining Methodology in EATM Knowledge Enrichment

To overcome the difficulty of deriving quantitative insights solely from the expertise, we utilized data mining methodologies to analyze the ancient medical literature *GYF*. However, specialized expertise is required for conducting the cluster and network analyses. For instance, we had to decide the number of clusters to perform a k-means cluster analysis. The number of clusters was determined based on the classifications by the EATM expert group as well as the hierarchical cluster analysis. We aimed to determine the most appropriate number and positions of initial centroids for the k-means cluster analysis by comparing the misclassification rates and silhouette indices. However, clusters generated under optimal conditions were not necessarily more useful. The extracted information from the analyses had to be assessed by experts, which is generally regarded as essential to data mining processes and to maximize the utility of the knowledge [27].

In addition, subtle differences between the information extracted from the experts and cluster analyses were noted. By comparing six sets of H-I subnetworks generated by the EATM expert group and k-means cluster analysis, common therapeutic indications were revealed despite the fact that cluster divisions were not entirely identical and that the misclassification rate varied. Heterogeneity was observed in one comparative set, both in the network reflecting the prior knowledge of the EATM expert group and in the network generated by the data points of prescriptions on the periphery of clusters. These prescriptions were less clearly defined due to their position on the cluster edges. For instance, the network of the ‘Early phase of the disease’ generated by cluster analysis could not incorporate the specialized knowledge regarding the unique efficacy of Da huang (H15). Furthermore, the EATM expert group distinguished abscess-treating prescriptions from diarrhea-treating prescriptions based on the knowledge of the pathological differences between abscess and diarrhea. However, the cluster analysis, set to be solely based on the similarity of constituent herbs, grouped them into a single cluster, clearly and repeatedly presenting that abscess-treating prescriptions and diarrhea-treating prescriptions were related. Overall, EATM expertise can maximize the usefulness of the information derived from analyses by incorporating qualitative knowledge into data mining, while data mining techniques can help minimize biases that may arise from human prejudice and efficiently extract intuitive information from vast amounts of data.

### 4.4. Strengths and Limitations

IBD has been treated with various prescriptions to date in the clinical and research fields [5]. Although IBD treatment in EATM has been guided by the Carbuncle Theory, no quantitative rules for herbal prescriptions have been established. *GYF*, being the earliest existing specialized surgical text, has significantly influenced the development of the Carbuncle Theory and its clinical applications. A study has explored the rules of combining medicinal herbs of the prescriptions in *GYF* through association rule mining [10]. Moreover, research has been conducted on the characteristics of herbal medicines used in IBD treatments based on the literature published in the China National Knowledge Infrastructure from 2000 to 2020 [28]. However, research analyzing the rules of herb combinations used in IBD or carbuncle treatment, particularly in conjunction with symptoms and signs, to determine the therapeutic objectives of each combination is lacking. This study aimed to analyze the prescriptions and indications in prescriptions in *GYF*, utilizing data mining in combination with expertise to determine which specific herbal combinations were used for certain symptoms and signs.

This study has several limitations. First, the study used the frequency of appearance of symptoms and signs and herbal names in the text as indicators of importance, which may have introduced potential bias in the interpretation of results. However, the high frequency of appearance does not necessarily indicate their high importance in the therapeutic principles. For example, herbs such as Da huang (H15) and Bai jiang (H65) may have lower frequencies but could be crucial in specific prescriptions, owing to their individual efficacy and therapeutic goals. To reduce this potential bias, we constructed a subnetwork of each cluster to identify major combinations of indications and applied herbs, minimizing the dependence on frequency. Second, because of the small size and heterogeneous density of the dataset, applying the k-means clustering algorithm could result in an error. In this study, we conducted a sensitivity analysis on the k-means clustering algorithm and calculated the rate of misclassification and the silhouette index to assess the impact of the number and the positions of initial centroids on the clustering outcomes. Third, we excluded the dosage of herbs from the analysis because of the difficulties in the standardization of the measurement unit used throughout *GYF*. Although analyzing the therapeutic principle without considering the dosage of herbs is possible to some extent, the dosage may still serve as a critical effect modifier. Fourth, the treatment strategies in *GYF* reflect clinical knowledge from a specific era, which may have evolved over time and may not fully represent modern clinical knowledge. However, modern clinical knowledge originates from *GYF*, and quantitative approaches are severely lacking. Our research findings contribute significantly to addressing this gap. There is a need for future research to analyze texts on abscess and carbuncle treatment from the Tang, Song, Jin-Yuan, Ming, and Qing dynasties up to the modern era, using both quantitative and qualitative methods, to reveal knowledge evolution since *GYF* and extract insights applicable to current clinical practice and research.

## 5. Conclusions

This study utilized data mining methodologies with EATM expertise to analyze the therapeutic principles embedded in prescriptions in *GYF*, the earliest existing surgical text on Carbuncle Theory, extracting and visualizing tacit knowledge of treatment modalities for IBD. The abscess status and the patient’s healing capacity and related herbs were the main considerations in the carbuncle treatment, contrasting the conventional standard treatment. Considering the limitations of this study mentioned earlier, readers should critically interpret the results. Future research is needed to develop a comprehensive theoretical framework through the analysis of various EATM texts across different eras, followed by preclinical and clinical validation.

## Figures and Tables

**Table 1:** Cluster-specific herbs and indications derived from the comparison of expert group classification and k-means cluster analysis results. Subsets 1 through 6 are cluster comparison pairs generated using the expert group classification results and k-means cluster analysis. The prescription IDs, indications, and herbs that constitute each comparison pair in the subsets are presented in Tables S11–S16.

	Common Major Indications *	Common Major Herbs **
Subset 1	Diarrhea Coldness Abscess Abscess ruptured	H1 Gan cao, H10 Jie geng, H12 Dan gui, H25 Bai tou weng, H27 Bai shi zhi, H31 Fu zi, H46 Long gu, H49 ren shen, H69 Huang lian
Subset 2	The early phase of the disease Difficulty in defecation Difficulty in urination Fever	H1 Gan cao, H12 Dan gui, H15 Da huang, H42 Sheng ma, H67 Huang qin
Subset 3	Abscess ruptured Deficiency Fever	H1 Gan cao, H4 Gan di huang, H14 Da zao, H19 Mai dong, H30 Fu ling, H36 Sheng jiang, H49 Ren shen, H68 Huang qi
Subset 4	Fever Deficiency Thirst	H1 Gan cao, H12 Dang gui, H30 Fu ling, H37 Sheng di huang, H49 Ren shen, H50 Shao yao, H55 Dan zhu ye, H64 Tong cao, H67 Huang qin, H68 Huang qi
Subset 5	After laxation Difficulty in urination Fever	H1 Gan cao, H6 Gui xin, H12 Dang gui, H19 Mai dong, H41 Xiao mai, H49 Ren shen, H50 Shao yao, H55 Dan zhu ye, H67 Huang qin
Subset 6	Abscess unruptured Abscess Abscess ruptured	H7 Gua lou, H50 Shao yao, H52 Chi xiao dou, H58 Chuan xiong, H68 Huang qi

* ‘Common major indications’ are indications that were frequently identified in the compared pairs. ** ‘Common Major Herbs’ are herbs that were frequently identified in the compared pairs.

## Data Availability

The datasets supporting the conclusions of this article are included within the article and its Supplementary Materials.

## Supplementary Materials

The following supporting information can be downloaded at: https://www.mdpi.com/article/10.3390/healthcare12151499/s1, Table S1. List of all prescriptions in *Liu Juan Zi Gui Yi Fang* (*GYF*) with their IDs, major indications, and routes of administration. Table S2. Constituent herbs and indications of carbuncle treatment prescriptions, with prescription ID and pinyin name. Table S3. List of herbs used in carbuncle treatment prescriptions. Table S4. Standardized and tokenized therapeutic indications of selected prescriptions with indication ID. Table S5. Expert group classification of carbuncle treatment prescriptions: Diarrhea treatment prescriptions (E1). Column order arranged for easy herb similarity comparison. Table S6. Expert group classification of carbuncle treatment prescriptions: Abscess treatment prescriptions (E2). Column order arranged for easy herb similarity comparison. Table S7. Expert group classification of carbuncle treatment prescriptions: Da huang-including prescriptions (E3). Column order arranged for easy herb similarity comparison. Table S8. Expert group classification of carbuncle treatment prescriptions: Tonifying prescriptions (E4). Column order arranged for easy herb similarity comparison. E4-1, Huang qi tang fang group; E4-2, Sheng di huang tang fang group; E4-3, Zhu ye tang fang group. Table S9. Sensitivity analysis: comparison of k-means cluster analysis results using a seed of 12345 and varying the number of initial centroids from three to eight. Table S10. Sensitivity analysis: comparison of k-means cluster analysis results with varying positions of six initial centroids. Table S11. Herb–Indication (H-I) network of Subset 1: Diarrhea–Coldness–Abscess–Abscess ruptured. Table S12. Herb–Indication (H-I) network of Subset 2: Early phase of the disease–Difficulty in defecation–Difficulty in urination–Fever. Table S13. Herb–Indication (H-I) network of Subset 3: Abscess ruptured–Deficiency–Fever. Table S14. Herb–Indication (H-I) network of Subset 4: Fever–Deficiency–Thirst. Table S15. Herb–Indication (H-I) network of Subset 5: After relaxation–Difficulty in urination–Fever. Table S16. Herb–Indication (H-I) network of Subset 6: Abscess unruptured–Abscess–Abscess ruptured.

## Abbreviations

EATM: East Asian traditional medicine; *GYF*, *Liu Juan Zi Gui Yi Fang*; H-I, herb–indication; IBD, inflammatory bowel disease; ID, identifier.
