# Supplementary material for: Exploration of Therapeutic Strategies of Herbal Prescriptions for Carbuncle Treatment to Suggest Modern Approaches to Inflammatory Bowel Disease: Cluster and Network Analyses of the Book «Liu Juan Zi Gui Yi Fang»
Source: Healthcare (Basel). 2024 Jul 28;12(15):1499. doi: 10.3390/healthcare12151499 (PMC11311748; doi:10.3390/healthcare12151499)
Supplement: Supplementary file 1 [file healthcare-12-01499-s001.zip › healthcare-3073575-supplementary.pdf]

List of Tables

Table s1. List of all prescriptions in <Liu Juan Zi Gui Yi Fang (GYF)> with their IDs, major indications, and routes of administration.
Table s2. Constituent herbs and indications of carbuncle treatment prescriptions, with prescription ID and pinyin name.
Table s3. List of herbs used in carbuncle treatment prescriptions.
Table s4. Standardized and tokenized therapeutic indications of selected prescriptions with indication ID.
Table s5. Expert group classification of carbuncle treatment prescriptions: Diarrhea treatment prescriptions (E1). Column order arranged for easy herb similarity comparison.
Table s6. Expert group classification of carbuncle treatment prescriptions: Abscess treatment prescriptions (E2). Column order arranged for easy herb similarity comparison.
Table s7. Expert group classification of carbuncle treatment prescriptions: Da huang-including prescriptions (E3). Column order arranged for easy herb similarity comparison.
Table s8. Expert group classification of carbuncle treatment prescriptions: Tonifying prescriptions (E4). Column order arranged for easy herb similarity comparison. E4-1, Huang qi tang fang group; E4-2, Sheng di huang tang fang group; E4-3, Zhu ye tang fang group.
Table s9. Sensitivity analysis: comparison of k-means cluster analysis results using a seed of 12345 and varying the number of initial centroids from 3 to 8.
Table s10. Table s10. Sensitivity analysis: comparison of k-means cluster analysis results with varying positions of six initial centroids.
Table s11. Herb-Indication (H-I) network of Subset 1: Diarrhea–Coldness–Abscess–Abscess ruptured.
Table s12. Herb-Indication (H-I) network of Subset 2: Early phase of the disease–Difficulty in defecation–Difficulty in urination–Fever.
Table s13. Herb-Indication (H-I) network of Subset 3: Abscess ruptured–Deficiency–Fever.
Table s14. Herb-Indication (H-I) network of Subset 4: Fever–Deficiency–Thirst.
Table s15. Herb-Indication (H-I) network of Subset 5: After relaxation–Difficulty in urination–Fever.
Table s16. Herb-Indication (H-I) network of Subset 6: Abscess unruptured–Abscess–Abscess ruptured.

Table s1. List of all prescriptions in <Liu Juan Zi Gui Yi Fang (GYF)> with their IDs, major indications, and routes of administration.

Chapter of <GYF>	Original prescription name	Prescription ID	Original major indication	Major Indication	Route of administration
2	止血散方	P-1	金瘡	Trauma	Topical
2	蝙蝠消血散方	P-2	金瘡	Trauma	Oral
2	蒲黃散方	P-3	金瘡	Trauma	Oral
2	白蘞散方	P-4	金瘡	Trauma	Oral
2	小麥飲噴瘡方	P-5	金瘡	Trauma	topical
2	磁石散方	P-6	金瘡	Trauma	Oral
2	白芷散方	P-7	金瘡	Trauma	Oral
2	消石散方	P-8	金瘡	Trauma	Oral
2	止痛當歸散方	P-9	金瘡	Trauma	Oral
2	琥珀散方	P-10	金瘡	Trauma	Oral
2	敗弩散方	P-11	金瘡	Trauma	Oral
2	蛇銜散方	P-12	金瘡	Trauma	Oral
2	續斷散方	P-13	金瘡	Trauma	Oral
2	麻黃散方	P-14	金瘡	Trauma	Oral
2	白薇散方	P-15	金瘡	Trauma	Oral
2	內補當歸散方	P-16	金瘡	Trauma	Oral
2	內補蓯蓉散方	P-17	金瘡	Trauma	Oral
2	澤蘭散方	P-18	金瘡	Trauma	Oral
2	黃芪散方	P-19	金瘡	Trauma	Oral
2	解毒藍子散方	P-20	金瘡	Trauma	Topical/Oral
2	內補瞿麥散方	P-21	金瘡	Trauma	Oral
2	蒲黃散方	P-22	被打	Trauma	Oral

Chapter of <GYF>	Original prescription name	Prescription ID	Original major indication	Major Indication	Route of administration
2	續斷生肌膏方	P-23	癰疽 金瘡	Carbuncle, Trauma	Topical
2	甘菊膏方	P-24	金瘡 癰疽	Carbuncle, Trauma	Topical
2	生肌膏方	P-25	金瘡 癰疽	Carbuncle, Trauma	Topical
2	烏鷄湯方	P-26	金瘡	Trauma	Oral
2	烏鷄湯方	P-27	金瘡	Trauma	Oral
2	桃核湯方	P-28	金瘡	Trauma	Oral
2	豚心湯方	P-29	金瘡	Trauma	Oral
2	生肉膏方	P-30	金瘡 癰疽	Carbuncle, Trauma	Topical
2	白馬蹄散方	P-31	被打	Trauma	Oral
3	大黃湯方	P-32	癰	Carbuncle	Oral
3	淡竹葉湯方	P-33	癰疽	Carbuncle	Oral
3	生地黃湯方	P-34	發背 發乳 癰疽	Carbuncle	Oral
3	淡竹葉湯方	P-35	發背 乳癰	Carbuncle	Oral
3	生地黃湯方	P-36	癰疽	Carbuncle	Oral
3	黃芪湯方	P-37	癰疽	Carbuncle	Oral
3	生地黃湯方	P-38	背	Carbuncle	Oral
3	黃芪湯方	P-39	癰疽	Carbuncle	Oral
3	五味竹葉湯方	P-40	癰疽	Carbuncle	Oral
3	遠志湯方	P-41	癰疽 發背 乳	Carbuncle	Oral
3	白石脂湯方	P-42	發背 乳	Carbuncle	Oral
3	竹葉湯方	P-43	發癰疽	Carbuncle	Oral
3	竹葉湯方	P-44	癰疽	Carbuncle	Oral
3	竹葉湯方	P-45	癰疽	Carbuncle	Oral

Chapter of <GYF>	Original prescription name	Prescription ID	Original major indication	Major Indication	Route of administration
3	兼味竹葉湯方	P-46	發背 癰 發乳	Carbuncle	Oral
3	白石脂湯方	P-47	發背	Carbuncle	Oral
3	內補黃芪湯方	P-48	發背	Carbuncle	Oral
3	生地黃湯方	P-49	癰疽	Carbuncle	Oral
3	黃芪湯方	P-50	發背	Carbuncle	Oral
3	枳實湯方	P-51	炎疽	Carbuncle	Oral
3	大黃湯	P-52	腸癰	Carbuncle of intestine	Oral
3	大黃湯方	P-53	疹	Rash of carbuncle	Oral
3	辛夷湯方	P-54	妬乳	Carbuncle	Oral
3	內補黃芪湯方	P-55	乳結腫 癰	Carbuncle	Oral
3	黃芪湯方	P-56	癰腫	Carbuncle	Oral
3	內補黃芪湯方	P-57	癰疽	Carbuncle	Oral
3	竹葉湯方	P-58	癰	Carbuncle	Oral
3	增損竹葉湯方	P-59	癰疽	Carbuncle	Oral
3	黃芪湯方	P-60	癰疽	Carbuncle	Oral
4	松脂貼方	P-61	癰疽	Carbuncle	Topical
4	松脂貼方	P-62	癰疽	Carbuncle	Topical
4	松脂貼腫方	P-63	癰疽	Carbuncle	Topical
4	升麻薄極冷方	P-64	癰疽	Carbuncle	Topical
4	白蘞薄方	P-65	癰	Carbuncle	Topical
4	猪膽薄方	P-66	癰疽	Carbuncle	Topical
4	黃芪湯方	P-67	癰腫	Carbuncle	Oral
4	黃芪湯方	P-68	癤	Carbuncle	Oral

Chapter of <GYF>	Original prescription name	Prescription ID	Original major indication	Major Indication	Route of administration
4	黃芪湯方	P-69	癰	Carbuncle	Oral
4	內補竹葉黃芪湯方	P-70	癰	Carbuncle	Oral
4	赤石脂湯方	P-71	下	Diarrhea (Symptom of carbuncle of intestine)	Oral
4	溫中湯方	P-72	下	Diarrhea (Symptom of carbuncle of intestine)	Oral
4	補胃附子湯方	P-73	下	Diarrhea (Symptom of carbuncle of intestine)	Oral
4	猪蹄湯洗方	P-74	癰瘡	Carbuncle	Topical
4	猪蹄湯方	P-75	癰疽	Carbuncle	Topical
4	治肘疽方	P-76	肘疽	Carbuncle	Topical
4	增損散方	P-77	癰疽	Carbuncle	Oral
4	木占斯散方	P-78	癰	Carbuncle	Oral
4	木占斯散方	P-79	發背 發房 腸癰	Carbuncle, Carbuncle of intestine	Oral
4	瞿麥散方	P-80	癰疽	Carbuncle	Oral
4	食惡肉散方	P-81	癰	Carbuncle	Topical
4	食惡肉散方	P-82	癰疽	Carbuncle	Topical
4	兌膏方	P-83	癰疽	Carbuncle	Topical
4	靑龍膏方	P-84	食肉	Polyp	Topical
4	生肉膏方	P-85	癰疽 金瘡	Carbuncle, Trauma	Topical
4	搨湯方	P-86	丹 癰疽	Carbuncle	Topical
5	生肉地黃膏方	P-87	癰疽	Carbuncle	Topical
5	生肉黃芪膏方	P-88	癰疽 瘡	Carbuncle, Sores	Topical
5	生肉膏方	P-89	發背 乳	Carbuncle	Topical
5	黃芩膏方	P-90	癰腫	Carbuncle	Topical
5	鷗脂膏方	P-91	癰疽	Carbuncle	Topical

Chapter of <GYF>	Original prescription name	Prescription ID	Original major indication	Major Indication	Route of administration
5	續斷生肉膏方	P-92	癰疽 金瘡	Carbuncle, Trauma	Topical
5	甜竹葉膏方	P-93	癰疽 瘡	Carbuncle, Sores	Topical
5	生肉茼草膏方	P-94	癰疽	Carbuncle	Topical
5	蛇銜膏方	P-95	癰疽	Carbuncle	Topical/Oral
5	食肉膏方	P-96	癰疽	Carbuncle	Topical
5	大黃食肉膏方	P-97	癰疽	Carbuncle	Topical
5	蘆茹散方	P-98	癰疽	Carbuncle	Topical
5	發瘡膏方	P-99	癰疽	Carbuncle	Topical
5	惡瘡膏方	P-100	疥癬	Scabies	Topical
5	五黃膏方	P-101	疥癬 惡瘡	Scabies, Sores	Topical
5	水銀膏方	P-102	疥癬 惡瘡	Scabies, Sores	Topical
5	麝香膏方	P-103	面皯皰	Pustule	Topical
5	木蘭膏方	P-104	面皻皰	Pustule	Topical
5	鸕鷀屎膏方	P-105	皻皰	Pustule	Topical
5	生髮白芷膏方	P-106	髮頹	Hair loss	Topical
5	丹參膏方	P-107	治婦人乳腫痛	Carbuncle	Topical
5	五味子膏方	P-108	頭白頹瘡	Hair loss with scalp sore	Topical
5	膏方	P-109	疽瘻瘑疥諸惡瘡	Carbuncle, Fistula, Scabies, Sores	Topical
5	冶葛膏方	P-110	瘑疽諸瘡	Carbunle, Sores	Topical
5	丹砂膏方	P-111	傷寒 溫毒 熱疾 鼻塞 耳聾 寒癖 霍亂 眼中風 等	Contagious diseases, Nasal congestion, Deafness, Cold intolerance, Enteritis, Opthalmic diseases, etc.	Topical/Oral
5	丹砂膏方(又方)	P-112			Topical/Oral
5	丹砂膏方(又方)	P-113			Topical/Oral
5	赤膏治百病方	P-114	傷寒 癰疽 痹 等	Contagious diseases, Carbuncle, Paralysis, etc.	Topical

Chapter of <GYF>	Original prescription name	Prescription ID	Original major indication	Major Indication	Route of administration
5	丹妙膏方	P-115	瘭疽	Carbuncle	Oral
5	麝香膏方	P-116	瘭疽	Carbuncle	Topical
5	生芎藭膏方	P-117	疔腫	Carbuncle	Topical
5	丹砂膏方	P-118	瘭疽	Carbuncle	Oral
5	丹砂膏方	P-119	風溫 瘑疽 諸惡瘡	Contagious diseases, Carbuncle, Sores	Topical
5	丹砂膏方	P-120	瘑疥 癬 諸惡瘡	Scabies, Fungal infections, Sores	Topical/Oral
5	紫草膏方	P-121	小兒豆瘡	Scalp sores of children	Topical
5	水銀膏方	P-122	小兒熱瘡	Fever rashes of children	Topical
5	柏皮膏方	P-123	火瘡	Burn	Topical
5	羊髓膏方	P-124	瘭疽	Carbuncle	Topical
5	升麻膏方	P-125	腫瘡	Sores	Topical
5	生地黃膏方	P-126	熱瘡	Sores	Topical
5	雄黃膏方	P-127	惡瘡	Sores	Topical
5	水銀膏方	P-128	瘑 疽 瘻	Carbuncle, Fistula	Topical
5	白蘞膏方	P-129	疿 瘰癧瘡	Heat rashes, Scrofula	Topical
5	白蘞膏方	P-130	皮膚中熱疿 瘰癧	Heat rashes, Scrofula	Topical
5	生地黃膏方	P-131	熱瘡	Sores	Topical
5	生地黃膏方	P-132	熱瘡	Sores	Topical
5	黃連膏方	P-133	濕熱諸瘡	Sores	Topical
5	蛇床子膏方	P-134	熱瘡	Sores	Topical
5	木蘭膏方	P-135	熱瘡	Sores	Topical
5	黃連膏方	P-136	熱瘡	Sores	Topical
5	甘草膏方	P-137	灸瘡	Burn	Topical

Chapter of <GYF>	Original prescription name	Prescription ID	Original major indication	Major Indication	Route of administration
5	黃芪膏方	P-138	癰	Carbuncle	Topical
5	白芷摩膏方	P-139	癰疽	Carbuncle	Topical
5	治諸疽瘡膏方	P-140	疽瘡	Carbuncle, Sores	Topical
5	白芷膏方	P-141	鼻中塞	Nasal congestion	Topical
5	羊屎膏方	P-142	竹木所刺入手足	Thorn prick	Topical
5	朮膏方	P-143	湯潑入肉爛壞	Burn	Topical
5	柏皮膏方	P-144	火燒爛壞	Burn	Topical
5	又方	P-145			Topical
5	蘆茹膏方	P-146	瘑疽 疥癬 惡瘡	Carbuncle, Scabies, Sores	Topical
5	雌黃膏方	P-147	婦人妬乳	Carbuncle	Topical
5	麝香膏方	P-148	諸惡瘡	Sores	Topical
5	牛屎熏方	P-149	頭瘡 惡瘡 骨疽	Scalp sore, Sores, Carbuncle	Topical
5	六物滅瘢膏方	P-150	諸傷	Injuries	Topical
5	<小品>滅瘢方	P-151	*	*	Topical
5	又方	P-152			Topical

* The original description of indications for P-151 and P-152 is absent in <GYF>.

Table s2. Constituent herbs and indications of carbuncle treatment prescriptions, with prescription ID and pinyin name.

Prescription ID	Original prescription name	Prescription name Pinyin	Original prescription constituent herbs	Tokenized original therapeutic indications
P-32	大黃湯方	Da huang tang fang (DHTF)	大黃 梔子 升麻 黃芩 芒硝	大便不通, 小便不通, 熱
P-33	淡竹葉湯方	Dan zhu ye tang fang (DZYTF)	竹葉 瓜蔞 通草 前胡 升麻 茯苓 黃芩 知母 甘草 石膏 生地黃 芍藥 大黃 黃芪 當歸 人蔘	渴, 結實, 大便不通, 小便不通, 煩/悶, 吐, 寒熱
P-34	生地黃湯方	Sheng di huang tang fang (SDHTF)	生地黃 竹葉 黃芩 黃芪 甘草 茯苓 麥門冬 升麻 前胡 知母 芍藥 瓜蔞 大棗 當歸 人蔘	渴, 熱, 虛
P-35	淡竹葉湯方	Dan zhu ye tang fang (DZYTF)	竹葉 麥門冬 黃芪 芍藥 乾地黃 生薑 前胡 黃芩 升麻 遠志 瓜蔞 大棗 當歸	取利後
P-36	生地黃湯方	Sheng di huang tang fang (SDHTF)	生地黃 人蔘 甘草 黃芪 芍藥 茯苓 當歸 川芎 黃芩 通草 大棗 竹葉	熱, 虛
P-37	黃芪湯方	Huang qi tang fang (HQTF)	生地黃 竹葉 小麥 黃芪 黃芩 前胡 大黃 瓜蔞 通草 芍藥 升麻 茯苓 甘草 知母 人蔘 當歸	渴, 熱, 虛
P-38	生地黃湯方	Sheng di huang tang fang (SDHTF)	生地黃 人蔘 甘草 芍藥 通草 茯苓 黃芪 黃芩 竹葉 大棗 當歸 川芎	
P-39	黃芪湯方	Huang qi tang fang (HQTF)	黃芪 人蔘 甘草 芍藥 當歸 生薑 大棗 乾地黃 茯苓 白朮 遠志	虛
P-40	五味竹葉湯方	Wu wei zhu ye tang fang (WWZYTF)	竹葉 五味子 前胡 當歸 乾地黃 人蔘 小麥 黃芪 黃芩 麥門冬 生薑 甘草 升麻 大棗 桂心	
P-41	遠志湯方	Yuan zhi tang fang (YZTF)	遠志 當歸 甘草 桂心 川芎 黃芪 人蔘 麥門冬. 茯苓 乾地黃 生薑 大棗	少氣, 已潰/去膿, 虛
P-42	白石脂湯方	Bai shi zhi tang fang (BSZTF)	白石脂 龍骨 當歸 桔梗 女萎 黃連 甘草 白頭翁 乾薑	下
P-43	竹葉湯方	Zhu ye tang fang (ZYTF)	竹葉 小麥 乾地黃 人蔘 黃芩 前胡 升麻 麥門冬 生薑 黃芪 芍藥 大棗十 桂心 遠志 當歸 甘草	不食, 取利後
P-44	竹葉湯方	Zhu ye tang fang (ZYTF)	竹葉 小麥 乾地黃 黃芪 人蔘 甘草 芍藥 石膏 通草 升麻 黃芩 前胡 大棗 麥門冬	小便不通, 取利後

Prescription ID	Original prescription name	Prescription name Pinyin	Original prescription constituent herbs	Tokenized original therapeutic indications
P-45	竹葉湯方	Zhu ye tang fang (ZYTF)	竹葉 小麥 人蔘 黃芩 前胡 芍藥 甘草 乾地黃 當歸 桂心 黃芪 麥門冬 龍骨 牡蠣 赤蛸蟓 大棗	小便不通, 熱, 取利後
P-46	兼味竹葉湯方	Jian wei zhu ye tang fang (JWZYTF)	竹葉 小麥 黃芪 黃芩 五味子 人蔘 前胡 乾地黃 當歸 大棗 麥門冬 升麻 桂心 甘草 生薑	
P-47	白石脂湯方	Bai shi zhi tang fang (BSZTF)	白石脂 龍骨 當歸 桔梗 女萎 白頭翁 乾薑 黃連	已潰/去膿, 下
P-48	內補黃芪湯方	Nei bu huang qi tang fang (NBHQTF)	黃芪 乾地黃 人蔘 茯苓 當歸 芍藥 川芎 桂心 遠志 甘草 麥門冬 生薑 大棗	少氣, 已潰/去膿, 虛
P-49	生地黃湯方	Sheng di huang tang fang (SDHTF)	生地黃 人蔘 甘草 芍藥 茯苓 川芎 通草 黃芩 當歸 大棗 竹葉	熱, 虛
P-50	黃芪湯方	Huang qi tang fang (HQTF)	黃芪 黃芩 遠志 麥門冬 乾地黃 人蔘 川芎 甘草 芍藥 當歸 大棗 生薑 鷄肶胵 桑螵蛸	
P-51	枳實湯方	Zhi shi tang fang (ZSTF)	枳實 射干 升麻 乾地黃 黃芩 前胡 犀角 大黃 麝香	
P-52	大黃湯方	Da huang tang fang (DHTF)	大黃 牧丹 芥子 芒硝 桃仁	
P-53	大黃湯方	Da huang tang fang (DHTF)	大黃 梔子 升麻 黃芩 甘草	初期
P-54	辛夷湯方	Xin yi tang fang (XYTF)	辛夷 大棗 桂心 防風 白朮 甘草 生薑 澤蘭	
P-55	內補黃芪湯方	Nei bu huang qi tang fang (NBHQTF)	黃芪 茯苓 芍藥 麥門冬 甘草 厚朴 人蔘 生薑 乾地黃	熱, 已潰/去膿, 腫
P-56	黃芪湯方	Huang qi tang fang (HQTF)	黃芪 麥門冬 黃芩 梔子 芍藥 瓜蔞 乾地黃 升麻	熱, 腫
P-57	內補黃芪湯方	Nei bu huang qi tang fang (NBHQTF)	黃芪 茯苓 桂心 人蔘 甘草 生薑 當歸 五味子 大棗	熱, 已潰/去膿, 虛
P-58	竹葉湯方	Zhu ye tang fang (ZYTF)	竹葉 半夏 甘草 厚朴 小麥 生薑 當歸 麥門冬 人蔘 桂心 黃芩	滿, 上氣, 已潰/去膿, 虛
P-59	增損竹葉湯方	Zeng sun zhu ye tang fang (ZSZYTF)	竹葉 當歸 茯苓 人蔘 前胡 黃芩 桂心 芍藥 甘草 大棗 小麥 麥門冬	煩/悶, 熱, 腫,
P-60	黃芪湯方	Huang qi tang fang (HQTF)	黃芪 生薑 石膏末 甘草 芍藥 升麻 人蔘 知母 茯苓 桂心 麥門冬 大棗 乾地黃	熱, 已潰/去膿

Prescription ID	Original prescription name	Prescription name Pinyin	Original prescription constituent herbs	Tokenized original therapeutic indications
P-67	黃芪湯方	Huang qi tang fang (HQTF)	黃芪 瓜蔞 乾地黃 升麻 麥門冬 梔子 芍藥 黃芩	渴, 熱, 腫
P-68	黃芪湯方	Huang qi tang fang (HQTF)	黃芪 人蔘 川芎 當歸 甘草 遠志 乾地黃 大棗 生薑 麥門冬	熱
P-69	黃芪湯方	Huang qi tang fang (HQTF)	黃芪 甘草 桂心 芍藥 半夏 生薑 飴	未潰
P-70	內補竹葉黃芪湯方	Nei bu zhu ye huang qi tang fang (NBZYHQTF)	竹葉 黃芪 甘草 芍藥 黃芩 人蔘 桂心 大棗 乾地黃 升麻 茯苓 生薑	癰
P-71	赤石脂湯方	Chi shi zhi tang fang (CSZTF)	赤石脂 人蔘 甘草 乾薑 龍骨 附子	下, 寒冷
P-72	溫中湯方	Wen zhong tang fang (WZTF)	甘草 乾薑 附子 蜀椒	下, 寒冷
P-73	補胃附子湯方	Bu wei fu zi tang fang (BWFZTF)	附子 當歸 人蔘 黃連 甘草 乾薑 桂心 芍藥 蜀椒	下
P-77	增損散方	Zeng sun san fang (ZSSF)	黃芪 小豆 川芎 白斂 瓜蔞 芍藥	膿
P-78	木占斯散方	Mu zhan si san fang (MZSSF)	木占斯 桂心 人蔘 細辛 敗醬 乾薑 厚朴 甘草 防風 桔梗	膿
P-79	木占斯散方	Mu zhan si san fang (MZSSF)	木占斯 厚朴 甘草 細辛 瓜蔞 防風 乾薑 人蔘 桔梗 敗醬	
P-80	瞿麥散方	Qu mai san fang (QMSF)	瞿麥 白芷 黃芪 當歸 細辛 芍藥 薏苡仁 川芎 赤小豆	膿, 疼/痛, 未潰, 已潰/去膿
P-95	蛇銜膏方	She xian gao fang (SXGF)	蛇銜 大戟 大黃 芍藥 附子炮 當歸 獨活 芒硝 黃芩 細辛 川芎 蜀椒 薤白	膿
P-115	丹妙膏方	Dan miao gao fang (DMGF)	丹砂 犀角 射干 大黃 川芎 麝香 黃芩 生地黃 升麻 前胡 沈香 青木香	
P-118	丹砂膏方	Dan sha gao fang (DSGF)	丹砂 犀角 射干 生地黃 大黃 升麻 川芎 麝香 前胡 沈香 黃芩 青木香	初期

Table s3. List of herbs used in carbuncle treatment prescriptions.

Tokenized original name	Herb ID	Chinese name Pinyin	English name
甘草	H1	Gan cao (GC)	Licorice
芥子	H2	Jie zi (JZ)	Mustard seed
乾薑	H3	Gan jiang (GJ)	Ginger
乾地黃	H4	Gan di huang (GDH)	Dried rehmannia root
鷄肶胵	H5	Ji nei jin (JNJ)	Chicken gizzard lining gallus
桂心	H6	Gui xin (GX)	Cinnamon bark
栝樓	H7	Gua lou (GL)	Trichosanthes seed
飴	H8	Yi tang (YT)	Barley malt sugar
瞿麥	H9	Qu mai (QM)	Dianthus herb
桔梗	H10	Jie geng (JG)	Platycodon root
丹砂	H11	Zhu sha (ZS)	Cinnabar
當歸	H12	Dang gui (DG)	Angelica gigas root
大戟	H13	Jing da ji (JDJ)	Euphorbia
大棗	H14	Da zao (DZ)	Jujube
大黃	H15	Da huang (DH)	Rhubarb
桃仁	H16	Tao ren (TR)	Peach kernel
獨活	H17	Du huo (DH)	Aralia continentalis root
芒硝	H18	Mang xiao (MX)	Glauber's salt
麥門冬	H19	Mai dong (MD)	Liriope tuber
牡蠣	H20	Mu li (ML)	Oyster shell
牡丹皮	H21	Mu dan pi (MDP)	Moutan root bark
木占斯	H22	Mu zhan si (MZS)	*NA*[1]
半夏	H23	Ban xia (BX)	Pinellia tuber
防風	H24	Fang feng (FF)	Saposhnikovia root
白頭翁	H25	Bai tou weng (BTW)	Pulsatilla root
白蘞	H26	Bai lian (BL)	Japanese peppervine root
白石脂	H27	Bai shi zhi (BSZ)	*NA*
白芷	H28	Bai zhi (BZ)	Angelica dahurica root
白朮	H29	Bai zhu (BZ)	Atractylodes rhizome white
茯苓	H30	Fu ling (FL)	Poria
附子	H31	Fu zi (FZ)	Prepared aconite
射干	H32	She gan (SG)	Leopard lily
蛇銜	H33	She xian (SX)	*NA*
麝香	H34	She xiang (SX)	Musk
桑螵蛸	H35	Sang piao xiao (SP)	Mantis egg case
生薑	H36	Sheng jiang (SJ)	Raw ginger

[1] *NA*：not available.

Tokenized original name	Herb ID	Chinese name Pinyin	English name
生地黃	H37	Sheng di huang (SDH)	Raw rehmannia root
犀角	H38	Xi jiao (XJ)	Rhinoceros horn
石膏	H39	Shi gao (SG)	Gypsum fibrosum
細辛	H40	Xi xin (XX)	Asiasarum root and rhizome
小麥	H41	Xiao mai (XM)	Light wheat
升麻	H42	Sheng ma (SM)	Cimicifuga rhizome
辛夷	H43	Xin yi (XY)	Magnolia bud
女萎	H44	Nu wei (NW)	*NA*
五味子	H45	Wu wei zi (WWZ)	Schisandra fruit
龍骨	H46	Long gu (LG)	Os Draconis
遠志	H47	Yuan zhi (YZ)	Polygala root
薏苡仁	H48	Yi yi ren (YYR)	Coix seed
人蔘	H49	Ren shen (RS)	Ginseng
芍藥	H50	Shao yao (SY)	Peony root
赤石脂	H51	Chi shi zhi (CSZ)	Halloysite
赤小豆	H52	Chi xiao dou (CXD)	Red bean
赤蛸蟭	H53	Chi xiao tiao (CXT)	*NA*
前胡	H54	Qian hu (QH)	Hogfennel root
竹葉	H55	Dan zhu ye (DZY)	Lophatherum herb
知母	H56	Zhi mu (ZM)	Anemarrhena rhizome
枳實	H57	Zhi shi (ZS)	Poncirus immature fruit
川芎	H58	Chuan xiong (CX)	Cnidium rhizome
青木香	H59	Qing mu xiang (QMX)	*NA*
蜀椒	H60	Hua jiao (HJ)	Zanthoxylum peel
梔子	H61	Zhi zi (ZZ)	Gardenia fruit
沈香	H62	Chen xiang (CX)	Aloe wood
澤蘭	H63	Ze lan (ZL)	Lycopus herb
通草	H64	Tong cao (TC)	Ricepaperplant pith
敗醬	H65	Bai jiang (BJ)	Patrinia root
薤白	H66	Xie bai (XB)	Garlic chives
黃芩	H67	Huang qin (HQ)	Scutellaria root
黃芪	H68	Huang qi (HQ)	Astragalus root
黃連	H69	Huang lian (HL)	Coptis rhizome
厚朴	H70	Hou po (HP)	Magnolia bark

Table s4. Standardized and tokenized therapeutic indications of selected prescriptions with indication ID.

Indication ID	Tokenized therapeutic indication verbatim	Translation in English[#]
S1	渴	Thirst
S2	結實	Sign of lumped stools
S3	膿	Abscess
S4	大便不通	Difficulty in defecation
S5	疼/痛	Pain
S6	滿	Fullness
S7	未潰	Abscess unruptured
S8	煩/悶	Vexation/Oppression
S9	不食	Anorexia
S10	上氣	Qi reflux
S11	少氣	Weak or faint breathing
S12	小便不通	Difficulty in urination
S13	熱	Fever
S14	已潰/去膿	Abscess ruptured
S15	腫	Swelling
S16	初期	The early phase of the disease
S17	取利後	After laxation
S18	吐	Vomit
S19	下	Diarrhea
S20	寒/冷	Coldness
S21	寒熱	Aversion to cold with fever
S22	虛	Deficiency

[#] The indications verbatim were standardized and translated into English by consensus of the three researchers according to 「WHO international standard terminologies on traditional medicine in the Western Pacific Region」 (World Health Organization. Manila. WHO Regional Office for the Western Pacific; 2007), and the English edition of「Donguibogam」 (Heo Jun. Seoul, South Korea. Jinhan M&B; 2020).

Table s5. Expert group classification of carbuncle treatment prescriptions: Diarrhea treatment prescriptions (E1). Column order arranged for easy herb similarity comparison.

	Gan jiang	Gan cao	Fu zi	Long gu	Dagn gui	Huang lian	Bai shi zhi	Chi shi zhi	Ren shen	Jie geng	Nu wei	Hua jiao	Bai tou weng	Gui xin	Shao yao
	乾薑	甘草	附子	龍骨	當歸	黃連	白石脂	赤石脂	人蔘	桔梗	女萎	蜀椒	白頭翁	桂心	芍藥
	H3	H1	H31	H46	H12	H69	H27	H51	H49	H10	H44	H60	H25	H6	H50
P-42	○	○		○	○	○	○			○	○		○		
P-47	○			○	○	○	○			○	○		○		
P-71	○	○	○	○				○	○						
P-72	○	○	○									○			
P-73	○	○	○		○	○		○	○			○		○	○

Table s6. Expert group classification of carbuncle treatment prescriptions: Abscess treatment prescriptions (E2). Column order arranged for easy herb similarity comparison.

	Mu zhan si	Ren shen	Xi xin	Bai jiang	Gan jiang	Hou po	Gan cao	Fang feng	Jie geng	Gui xin	Gua lou
	木占斯	人蔘	細辛	敗醬	乾薑	厚朴	甘草	防風	桔梗	桂心	栝樓
	H22	H49	H40	H65	H3	H70	H1	H24	H10	H6	H7
P-78	○	○	○	○	○	○	○	○	○	○	
P-79	○	○	○	○	○	○	○	○	○		○

Table s7. Expert group classification of carbuncle treatment prescriptions: Da huang-including prescriptions (E3). Column order arranged for easy herb similarity comparison.

	Da huang	Huang qin	Sheng ma	Qian hu	Sheng di huang	Gan cao	Shao yao	Dang gui	Dan zhu ye	Gua lou	Tong cao	Fu ling	Zhi mu	Huang qi	Ren shen	Shi gao	Xiao mai	She gan	Xi jiao	She xiang	Chuan xiong	Zhu sha	Chen xiang	Qing mu xiang	Zhi zi	Mang xiao	Zhi shi	Gan di huang	She xian	Jing da ji	Fu zi	Du huo	Xi xin	Hua jiao	Xie bai	Mu dan pi	Jie zi	Tao ren
	大黃	黃芩	升麻	前胡	生地黃	甘草	芍藥	當歸	竹葉	栝樓	通草	茯苓	知母	黃芪	人蔘	石膏	小麥	射干	犀角	麝香	川芎	丹砂	沈香	青木香	梔子	芒硝	枳實	乾地黃	蛇銜	大戟	附子	獨活	細辛	蜀椒	薤白	牡丹皮	芥子	桃仁
	H15	H67	H42	H54	H37	H1	H50	H21	H55	H7	H64	H30	H56	H68	H49	H39	H41	H32	H38	H34	H58	H11	H62	H59	H61	H18	H57	H4	H33	H13	H31	H17	H40	H60	H66	H21	H2	H16
P-32	○	○	○																						○	○												
P-53	○	○	○			○																			○													
P-33	○	○	○	○	○	○	○	○	○	○	○	○	○	○	○	○																						
P-37	○	○	○	○	○	○	○	○	○	○	○	○	○	○	○		○																					
P-51	○	○	○	○														○	○	○							○	○										
P-115	○	○	○	○	○													○	○	○	○	○	○	○														
P-118	○	○	○	○	○													○	○	○	○	○	○	○														
P-52	○																									○										○	○	○
P-95	○	○					○	○													○					○			○	○	○	○	○	○	○			

Table s8. Expert group classification of carbuncle treatment prescriptions: Tonifying prescriptions (E4). Column order arranged for easy herb similarity comparison. E4-1, Huang qi tang fang group; E4-2, Sheng di huang tang fang group; E4-3, Zhu ye tang fang group.

		Sheng di huang	Huang qi	Gan cao	Ren shen	Gan di huang	Shao yao	Dang gui	Sheng jiang	Da zao	Huang qin	Mai dong	Dan zhu ye	Fu ling	Chuan xiong	Qian hu	Gui xin	Sheng ma	Xiao mai	Gua lou	Yuan zhi	Tong cao	Zhi mu	Remainder[∀]
		生地黃	黃芪	甘草	人蔘	乾地黃	芍藥	當歸	生薑	大棗	黃芩	麥門冬	竹葉	茯苓	川芎	前胡	桂心	升麻	小麥	栝樓	遠志	通草	知母	
		H37	H68	H1	H49	H4	H50	H21	H36	H14	H67	H19	H55	H30	H58	H54	H6	H42	H41	H7	H47	H64	H56	
E4-1	P-39		○	○	○	○	○	○	○	○				○							○			Bai zhu (H29)
	P-41		○	○	○	○		○	○	○		○		○	○		○				○			
	P-48		○	○	○	○	○	○	○	○		○		○	○		○				○			
	P-50		○	○	○	○	○	○	○	○	○	○			○						○			Ji nei jin (H5), Sang piao xiao (H35)
	P-55		○	○	○	○	○		○			○		○										Hou po (H70)
	P-56		○			○	○				○	○						○		○				Zhi zi (H61)
	P-57		○	○	○			○	○	○		○		○			○				○			Wu wei zi (H45)
	P-60		○	○	○	○	○		○	○		○		○			○	○					○	
	P-67		○			○	○				○	○						○		○				Zhi zi (H61)
	P-68		○	○	○	○		○	○	○		○			○						○			
	P-69		○	○			○		○								○							Ban xia (H23), Yi tang (H8)
	P-70		○	○	○	○	○		○	○	○		○	○			○	○						
E4-2	P-33	○	○	○	○		○	○			○		○	○		○		○		○		○	○	Da huang (H15), Shi gao (H39)
	P-37	○	○	○	○		○	○			○		○	○		○		○	○	○		○	○	Da huang (H15)
	P-34	○	○	○	○		○	○		○	○	○	○	○		○		○		○			○	

[∀] Herbs that have appeared less than three times are recorded in this 'Remainder' column due to space constraints.

		Sheng di huang	Huang qi	Gan cao	Ren shen	Gan di huang	Shao yao	Dang gui	Sheng jiang	Da zao	Huang qin	Mai dong	Dan zhu ye	Fu ling	Chuan xiong	Qian hu	Gui xin	Sheng ma	Xiao mai	Gua lou	Yuan zhi	Tong cao	Zhi mu	Remainder∀
		生地黃	黃芪	甘草	人蔘	乾地黃	芍藥	當歸	生薑	大棗	黃芩	麥門冬	竹葉	茯苓	川芎	前胡	桂心	升麻	小麥	栝樓	遠志	通草	知母	
		H37	H68	H1	H49	H4	H50	H21	H36	H14	H67	H19	H55	H30	H58	H54	H6	H42	H41	H7	H47	H64	H56	
	P-36	○	○	○	○		○	○		○	○		○	○	○							○		
	P-38	○	○	○	○		○	○		○	○		○	○	○							○		
	P-49	○		○	○		○	○		○	○		○	○	○							○		
E4-3	P-35		○			○	○	○	○	○	○	○	○			○		○		○	○			
E4-3	P-43		○	○	○	○	○	○	○	○	○	○	○			○	○	○	○		○			
E4-3	P-44		○	○	○	○	○			○	○	○	○			○		○	○			○		Shi gao (H39)
E4-3	P-45		○	○	○	○	○	○		○	○	○	○			○	○		○					Long gu (H46), Mu li (H20), Chi xiao tiao (H53)
E4-3	P-40		○	○	○	○		○	○	○	○	○	○			○	○	○	○					We wei zi (H45)
E4-3	P-46		○	○	○	○		○	○	○	○	○	○			○	○	○	○					We wei zi (H45)
E4-3	P-58			○	○			○	○		○	○	○				○		○					Ban xia (H23), Hou po (H70)
E4-3	P-59			○	○		○	○		○	○	○	○	○		○	○		○					
E4-4	P-54			○					○	○							○							Bai zhu (H29), Xin yi (H43), Fang feng (H24), Ze lan (H63)
E4-4	P-77		○				○								○					○				Chi shi zhi (H51), Bai lian (H26)
E4-4	P-80		○				○	○							○									Bai zhi (H28), Xi xin (H40), Yi yi ren (H48), Chi xiao dou (H52), Qu mai (H9)

Table s9. Sensitivity analysis: comparison of k-means cluster analysis results using a seed of 12345 and varying the number of initial centroids from 3 to 8.

Prescription ID	k=3	k=4	k=5	k=6	k=7	k=8
P-32 大黃湯方	2	2	2	2	2	8
P-33 淡竹葉湯方	1	4	4	4	4	4
P-34 生地黃湯方	1	4	4	4	4	4
P-35 淡竹葉湯方	1	1	1	1	1	1
P-36 生地黃湯方	1	4	4	4	4	4
P-37 黃芪湯方	1	4	4	4	4	4
P-38 生地黃湯方	1	4	4	4	4	4
P-39 黃芪湯方	3	3	3	3	3	3
P-40 五味竹葉湯方	1	1	1	1	1	1
P-41 遠志湯方	3	3	3	3	3	3
P-42 白石脂湯方	2	2	2	6	7	2
P-43 竹葉湯方	1	1	1	1	1	1
P-44 竹葉湯方	1	1	1	1	1	1
P-45 竹葉湯方	1	1	1	1	1	1
P-46 兼味竹葉湯方	1	1	1	1	1	1
P-47 白石脂湯方	2	2	2	6	7	2
P-48 內補黃芪湯方	3	3	3	3	3	3
P-49 生地黃湯方	1	4	4	4	4	4
P-50 黃芪湯方	3	3	3	3	3	3
P-51 枳實湯方	2	2	2	2	2	8
P-52 大黃湯方	2	2	2	2	6	6
P-53 大黃湯方	2	2	2	2	2	8
P-54 辛夷湯方	2	2	2	6	6	6
P-55 內補黃芪湯方	3	3	3	3	3	3
P-56 黃芪湯方	1	1	5	5	5	5
P-57 內補黃芪湯方	3	3	3	3	3	3
P-58 竹葉湯方	1	1	1	1	1	1
P-59 增損竹葉湯方	1	1	1	1	1	1
P-60 黃芪湯方	3	3	3	3	3	3
P-67 黃芪湯方	1	1	5	5	5	5
P-68 黃芪湯方	3	3	3	3	3	3
P-69 黃芪湯方	2	2	5	5	6	6
P-70 內補竹葉黃芪湯方	1	1	1	1	1	1
P-71 赤石脂湯方	2	2	2	6	7	7
P-72 溫中湯方	2	2	2	6	7	7
P-73 補胃附子湯方	2	2	2	6	7	7
P-77 增損散方	2	2	5	5	6	6
P-78 木占斯散方	2	2	2	6	7	7
P-79 木占斯散方	2	2	2	6	7	7
P-80 瞿麥散方	2	2	5	5	6	6

Prescription ID	k=3	k=4	k=5	k=6	k=7	k=8
P-95 瞿麥散方	2	2	2	2	6	6
P-115 丹妙膏方	2	2	2	2	2	8
P-118 丹砂膏方	2	2	2	2	2	8
Misclassification[1] (%)	14.62	14.62	9.52	0.22	1.55	1.55
Silhouette index	0.11	0.14	0.18	0.18	0.24	0.19

1 The misclassification was defined as when a pair of prescriptions was classified into the same cluster while the value of the Jaccard coefficient between them was zero.

Table s10. Table s10. Sensitivity analysis: comparison of k-means cluster analysis results with varying positions of six initial centroids.

Prescription ID	seed=12345	seed=71	seed=123	seed=148	seed=371
P-32 大黃湯方	2	3	6	5	6
P-33 淡竹葉湯方	4	1	4	4	1
P-34 生地黃湯方	4	1	4	4	1
P-35 淡竹葉湯方	1	2	2	3	3
P-36 生地黃湯方	4	1	4	4	1
P-37 黃芪湯方	4	1	4	4	1
P-38 生地黃湯方	4	1	4	4	1
P-39 黃芪湯方	3	5	1	2	2
P-40 五味竹葉湯方	1	2	2	3	3
P-41 遠志湯方	3	5	1	2	2
P-42 白石脂湯方	6	4	6	1	5
P-43 竹葉湯方	1	2	2	3	3
P-44 竹葉湯方	1	2	2	3	3
P-45 竹葉湯方	1	6	3	3	3
P-46 兼味竹葉湯方	1	2	2	3	3
P-47 白石脂湯方	6	4	6	1	5
P-48 內補黃芪湯方	3	5	1	2	2
P-49 生地黃湯方	4	1	4	4	1
P-50 黃芪湯方	3	5	1	2	2
P-51 枳實湯方	2	3	6	5	6
P-52 大黃湯方	2	3	6	5	6
P-53 大黃湯方	2	3	6	5	6
P-54 辛夷湯方	6	4	6	1	5
P-55 內補黃芪湯方	3	5	1	2	2
P-56 黃芪湯方	5	3	5	6	4
P-57 內補黃芪湯方	3	5	1	2	2
P-58 竹葉湯方	1	6	3	3	3
P-59 增損竹葉湯方	1	6	3	3	3
P-60 黃芪湯方	3	5	1	2	2
P-67 黃芪湯方	5	3	5	6	4
P-68 黃芪湯方	3	5	1	2	2
P-69 黃芪湯方	5	4	5	6	4
P-70 內補竹葉黃芪湯方	1	2	2	3	3
P-71 赤石脂湯方	6	4	6	1	5
P-72 溫中湯方	6	4	6	1	5
P-73 補胃附子湯方	6	4	6	1	5
P-77 增損散方	5	3	5	6	4
P-78 木占斯散方	6	4	6	1	5
P-79 木占斯散方	6	4	6	1	5
P-80 瞿麥散方	5	3	5	6	4

Prescription ID	seed=12345	seed=71	seed=123	seed=148	seed=371
P-95 瞿麥散方	2	3	6	5	6
P-115 丹妙膏方	2	3	6	5	6
P-118 丹砂膏方	2	3	6	5	6
Misclassification[1] (%)	0.22	2.66	9.52	0.22	0.22
Silhouette index	0.22	0.16	0.14	0.22	0.22

[1] The misclassification was defined as when a pair of prescriptions was classified into the same cluster while the value of the Jaccard coefficient between them was zero.

Table s11. Herb-Indication (H-I) network of Subset 1: Diarrhea–Coldness–Abscess–Abscess ruptured.

Indication	**Diarrhea – Coldness – Abscess - Abscess ruptured[1]**					
Subset 1	K-means cluster analysis			Expert group classification		
	P[2]	Major indications[3]	Herbs[4]	P	Indications	Herbs
	P-42	S19 Diarrhea	H1 Gan cao*	P-42	S19 Diarrhea	H1 Gan cao*
	P-47	S3 Abscess	H3 Gan jiang	P-47	S20 Coldness	H3 Gan jiang*
	P-54	S20 Coldness	H6 Gui xin	P-71	S14 Abscess ruptured	H6 Gui xin*
	P-71	S14 Abscess ruptured	H10 Jie geng*	P-72	S3 Abscess	H10 Jie geng*
	P-72		H12 Dang gui*	P-73		H12 Dang gui*
	P-73		H22 Mu zhan si	P-78		H22 Mu zhan si*
	P-78		H24 Fang feng	P-79		H24 Fang feng*
	P-79		H25 Bai tou weng*			H25 Bai tou weng*
			H27 Bai shi zhi*			H27 Bai shi zhi*
			H31 Fu zi*			H31 Fu zi*
			H40 Xi xin			H40 Xi xin*
			H44 Nu wei			H44 Nu wei*
			H46 Long gu*			H46 Long gu*
			H49 Ren shen*			H49 Ren shen*
			H50 Shao yao			H50 Shao yao
			H51 Chi shi zhi			H51 Chi shi zhi
			H60 Hua jiao			H60 Hua jiao
			H65 Bai jiang			H65 Bai jiang*
			H69 Huang lian*			H69 Huang lian*
			H70 Hou po			H70 Hou po*

1 Two subnetworks of 'Diarrhea treatment prescriptions (E1)' and 'Abscess treatment prescriptions (E2)' were included in the column of Expert group classification.
2 Prescriptions included in the cluster.
3 The ten most frequently identified indications were defined as the major indications.
4 All herbs comprising the network were listed.
* The ten most frequently constituted herbs in the subnetwork.

Table s12. Herb-Indication (H-I) network of Subset 2: Early phase of the disease–Difficulty in defecation–Difficulty in urination–Fever.

Indication	**The early phase of the disease – Difficulty in defecation – Difficulty in urination - Fever**							
Subset 2	K-means cluster analysis				Expert group classification			
	P[1]	Indications[2]	Herbs[3]		P	Indications	Herbs	
	P-32<br>P-51<br>P-52<br>P-53<br>P-95<br>P-115<br>P-118	S16 The early phase of the disease<br>S3 Abscess<br>S4 Difficulty in defecation<br>S12 Difficulty in urination<br>S13 Fever	H1 Gan cao*	H61 Zhi zi*	P-32<br>P-33<br>P-37<br>P-51<br>P-52<br>P-53<br>P-95<br>P-115<br>P-118	S1 Thirst<br>S4 Difficulty in defecation<br>S12 Difficulty in urination<br>S13 Fever<br>S16 The early phase of the disease<br>S2 Sign of lumped stools<br>S8 Vexation/Oppression<br>S21 Aversion to cold with fever<br>S22 Deficiency<br>S3 Abscess	H1 Gan cao*	H40 Xi xin
			H11 Zhu sha*	H62 Chen xiang*			H2 Jie zi	H41 Xiao mai
			H12 Dang gui*	H66 Xie bai			H4 Gan di huang	H42 Sheng ma*
			H13 Jing da ji*	H67 Huang qin*			H7 Gua lou*	H49 Ren shen
			H15 Da huang*				H11 Zhu sha	H50 Shao yao*
			H17 Du huo				H12 Dang gui*	H54 Qian hu*
			H18 Mang xiao*				H13 Jing da ji	H55 Dan zhu ye
			H31 Fu zi				H15 Da huang*	H56 Zhi mu
			H32 She gan				H16 Tao ren	H57 Zhi shi
			H33 She xian				H17 Du huo	H58 Chuan xiong
			H34 She xiang				H18 Mang xiao	H59 Qing mu xiang
			H37 Sheng di huang				H21 Mu dan pi	H60 Hua jiao
			H38 Xi jiao				H30 Fu ling*	H61 Zhi zi
			H40 Xi xin				H31 Fu zi	H62 Chen xiang
			H42 Sheng ma*				H32 She gan	H64 Tong cao
			H50 Shao yao				H33 She xian	H66 Xie bai
			H54 Qian hu				H34 She xiang	H67 Huang qin*
			H58 Chuan xiong				H37 Sheng di huang*	H68 Huang qi
			H59 Qing mu xiang				H38 Xi jiao	
			H60 Hua jiao				H39 Shi gao	

[1] Prescriptions included in the cluster.
[2] The ten most frequently identified indications were defined as the major indications.
[3] All herbs comprising the network were listed.
* The ten most frequently constituted herbs in the subnetwork.

Table s13. Herb-Indication (H-I) network of Subset 3: Abscess ruptured–Deficiency–Fever.

Indication	**Abscess ruptured – Deficiency - Fever**						
Subset 3	K-means cluster analysis			Expert group classification			
	P[1]	Indications[2]	Herbs[3]	P	Indications	Herbs	
	P-39	S14 Abscess ruptured	H1 Gan cao*	P-39	S13 Fever	H1 Gan cao*	H55 Dan zhu ye
	P-41	S22 Deficiency	H4 Gan di huang*	P-41	S14 Abscess ruptured	H4 Gan di huang*	H56 Zhi mu
	P-48	S13 Fever	H6 Gui xin*	P-48	S22 Deficiency	H5 Ji nei jin	H58 Chuan xiong
	P-50	S11 Weak or faint breathing	H12 Dang gui*	P-50	S11 Weak or faint breathing	H6 Gui xin*	H61 Zhi zi
	P-55	S15 Swelling	H14 Da zao*	P-55	S15 Swelling	H7 Gua lou	H67 Huang qin
	P-57		H19 Mai dong*	P-56	S1 Thirst	H8 Yi tang	H68 Huang qi*
	P-60		H29 Bai zhu	P-57	S7 Abscess unruptured	H12 Dang gui	H70 Hou po
	P-68		H30 Fu ling*	P-60		H14 Da zao*	
			H36 Sheng jiang*	P-67		H19 Mai dong*	
			H37 Sheng di huang	P-68		H23 Ban xia	
			H42 Sheng ma	P-69		H29 Bai zhu	
			H45 Wu wei zi	P-70		H30 Fu ling*	
			H47 Yuan zhi			H35 Sang piao xiao	
			H49 Ren shen*			H36 Sheng jiang*	
			H50 Shao yao			H39 Shi gao	
			H56 Zhi mu			H42 Sheng ma	
			H58 Chuan xiong			H45 Wu wei zi	
			H68 Huang qi*			H47 Yuan zhi	
			H70 Hou po			H49 Ren shen*	
						H50 Shao yao*	

[1] Prescriptions included in the cluster.
[2] The ten most frequently identified indications were defined as the major indications.
[3] All herbs comprising the network were listed.
* The ten most frequently constituted herbs in the subnetwork.

Table s14. Herb-Indication (H-I) network of Subset 4: Fever–Deficiency–Thirst.

Indication	**Fever – Deficiency - Thirst**					
Subset 4	K-means cluster analysis			Expert group classification		
	P[1]	Indications[2]	Herbs[3]	P	Indications	Herbs
	P-33	S13 Fever	H1 Gan cao*	P-33	S13 Fever	H1 Gan cao*
	P-34	S22 Deficiency	H7 Gua lou	P-34	S22 Deficiency	H7 Gua lou
	P-36	S1 Thirst	H12 Dang gui*	P-36	S1 Thirst	H12 Dang gui*
	P-37	S2 Sign of lumped stools	H14 Da zao	P-37	S2 Sign of lumped stools	H14 Da zao
	P-38	S4 Difficulty in defecation	H15 Da huang	P-38	S4 Difficulty in defecation	H15 Da huang
	P-49	S8 Vexation/Oppression	H19 Mai dong	P-49	S8 Vexation/Oppression	H19 Mai dong
		S12 Difficulty in urination	H30 Fu ling*		S12 Difficulty in urination	H30 Fu ling*
		S18 Vomit	H37 Sheng di huang*		S18 Vomit	H37 Sheng di huang*
		S21 Aversion to cold with fever	H39 Shi gao		S21 Aversion to cold with fever	H39 Shi gao
			H41 Xiao mai			H41 Xiao mai
			H42 Sheng ma			H42 Sheng ma
			H49 Ren shen*			H49 Ren shen*
			H50 Shao yao*			H50 Shao yao*
			H54 Qian hu			H54 Qian hu
			H55 Dan zhu ye*			H55 Dan zhu ye*
			H56 Zhi mu			H56 Zhi mu
			H58 Chuan xiong			H58 Chuan xiong
			H64 Tong cao*			H64 Tong cao*
			H67 Huang qin*			H67 Huang qin*
			H68 Huang qi*			H68 Huang qi*

[1] Prescriptions included in the cluster.
[2] The ten most frequently identified indications were defined as the major indications.
[3] All herbs comprising the network were listed.
* The ten most frequently constituted herbs in the subnetwork.

Table s15. Herb-Indication (H-I) network of Subset 5: After relaxation–Difficulty in urination–Fever.

Indication	**After laxation - Difficulty in urination - Fever**							
Subset 5	K-means cluster analysis				Expert group classification			
	P[1]	Indications[2]	Herbs[3]		P	Indications	Herbs	
	P-35	S17 After laxation	H1 Gan cao*	H55 Dan zhu ye*	P-35	S17 After laxation	H1 Gan cao*	H55 Dan zhu ye*
	P-40	S12 Difficulty in urination	H4 Gan di huang	H64 Tong cao	P-40	S12 Difficulty in urination	H4 Gan di huang	H64 Tong cao
	P-43	S13 Fever	H6 Gui xin*	H67 Huang qin*	P-43	S13 Fever	H6 Gui xin*	H67 Huang qin*
	P-44	S9 Anorexia	H7 Gua lou	H68 Huang qi	P-44	S9 Anorexia	H7 Gua lou	H68 Huang qi
	P-45	S8 Vexation/Oppression	H12 Dang gui*	H70 Hou po	P-45	S8 Vexation/Oppression	H12 Dang gui*	H70 Hou po
	P-46	S15 Swelling	H14 Da zao*		P-46	S15 Swelling	H14 Da zao*	
	P-58	S6 Fullness	H19 Mai dong*		P-58	S6 Fullness	H19 Mai dong*	
	P-59	S10 Qi reflux	H20 Mu li		P-59	S10 Qi reflux	H20 Mu li	
	P-70	S14 Abscess ruptured	H23 Ban xia			S14 Abscess ruptured	H30 Fu ling	
		S22 Deficiency	H30 Fu ling			S22 Deficiency	H36 Sheng jiang	
			H36 Sheng jiang				H39 Shi gao	
			H39 Shi gao				H41 Xiao mai*	
			H41 Xiao mai*				H42 Sheng ma	
			H42 Sheng ma				H45 Wu wei zi	
			H46 Long gu				H46 Long gu	
			H47 Yuan zhi				H47 Yuan zhi	
			H49 Ren shen*				H49 Ren shen*	
			H50 Shao yao*				H50 Shao yao*	
			H53 Chi xiao tiao				H53 Chi xiao tiao	
			H54 Qian hu				H54 Qian hu*	

1 Prescriptions included in the cluster.
2 The ten most frequently identified indications were defined as the major indications.
3 All herbs comprising the network were listed.
* The ten most frequently constituted herbs in the subnetwork.

Table s16. Herb-Indication (H-I) network of Subset 6: Abscess unruptured–Abscess–Abscess ruptured.

Indication	**Abscess unruptured - Abscess - Abscess ruptured**					
Subset 6	K-means cluster analysis			Expert group classification		
	P[1]	Indications[2]	Herbs[3]	P	Indications	Herbs
	P-56<br>P-67<br>P-69<br>P-77<br>P-80	S7 Abscess unruptured<br>S13 Fever<br>S15 Swelling<br>S3 Abscess<br>S5 Pain<br>S14 Abscess ruptured<br>S1 Thirst	H1 Gan cao	P-54<br>P-77<br>P-80	S3 Abscess<br>S5 Pain<br>S7 Abscess unruptured<br>S14 Abscess ruptured	H7 Gua lou*
			H4 Gan di huang*			H9 Qu mai*
			H6 Gui xin			H12 Dang gui*
			H7 Gua lou*			H26 Bai lian*
			H8 Yi tang			H28 Bai zhi*
			H9 Qu mai			H40 Xi xin*
			H12 Dang gui			H48 Yi yi ren*
			H19 Mai dong*			H50 Shao yao*
			H23 Ban xia			H52 Chi xiao dou*
			H26 Bai lian			H58 Chuan xiong*
			H28 Bai zhi			H68 Huang qi*
			H36 Sheng jiang			
			H40 Xi xin			
			H42 Sheng ma*			
			H48 Yi yi ren			
			H50 Shao yao*			
			H52 Chi xiao dou*			
			H58 Chuan xiong*			
			H61 Zhi zi*			
			H67 Huang qin*			
			H68 Huang qi*			

[1] Prescriptions included in the cluster.
[2] The ten most frequent identified indications were defined as the major indications.
[3] All herb comprising the network were listed.
* The ten most frequently constituted herbs in the subnetwork.
